# Indications and findings of upper gastrointestinal endoscopy in patients presenting to a District Hospital, Ghana

**DOI:** 10.11604/pamj.2019.34.82.18002

**Published:** 2019-10-11

**Authors:** Adwoa Agyei-Nkansah, Amoako Duah, Maite Alfonso

**Affiliations:** 1 Department of Medicine and Therapeutics School of Medicine and Dentistry, College of Health Science, University of Ghana, P.O.BOX 4236, Korle-Bu, Accra, Ghana; 2 St. Dominic Hospital, P.O.BOX 59, Akwatia, Ghana

**Keywords:** Upper gastrointestinal, endoscopy, dyspepsia, Ghana

## Abstract

**Introduction:**

Upper gastrointestinal (UGI) symptoms are among the commonest complaints for which patients seek medical attention. Characteristics of patients undergoing UGI endoscopy (UGIE) at the district hospital in Ghana are largely unknown. This study was to document the demographic characteristics, indications and endoscopic findings of patients undergoing UGIE at the district hospital in Ghana.

**Methods:**

This study used a cross-sectional design to consecutively recruit 371 patients referred to the Endoscopy Unit of the St. Dominic Hospital, Akwatia for UGIE. Demographic data and indications for the UGIE were recorded. Endoscopic findings per each participant were recorded. *Helicobacter pylori* (*H. pylori*) infection was confirmed by rapid-urease examination of gastric antral and body biopsies at endoscopy.

**Results:**

There were 159(42.9%) males out of the 371 patients. The age ranged from 4 to 94 years with a median age of 46 years. Dyspepsia was the commonest indication occurring in 282(76.0%) patients. The commonest endoscopic diagnosis was gastritis which occurred in 261(70.4%) patients. The prevalence of *H. pylori* obtained by immediate rapid-urease-campylobacter like- organism (CLO) test was 44.9%.

**Conclusion:**

The main indication for UGIE in the studied patients was dyspepsia and most of these patients had gastritis on endoscopy. Only few patients had normal findings. The prevalence of *H. pylori* in this population was low compared with most of the previous studies done in the country. There is the need to establish more endoscopy centres within the district hospitals in the country and more health professionals trained to perform them.

## Introduction

Upper Gastrointestinal (UGI) symptoms are among the commonest complaints for which patients seek medical attention, with the annual prevalence of dyspepsia approximating 25% [1]. Diseases associated with dyspepsia are leading causes of gastrointestinal morbidity and mortality globally. Peptic ulcer disease, gastroesophageal reflux disease and cancers affect millions of people worldwide [2]. Gastrointestinal endoscopy (UGIE) is one of the most commonly performed endoscopic procedures and provides valuable information in patients with gastroduodenal disorders. It gives a better diagnostic yield over radiology particularly in the investigation of upper gastrointestinal bleeding, inflammatory conditions of the UGI track like esophagitis, gastritis and duodenitis as well as the diagnosis of Mallory Weiss tears and vascular malformations [3]. Appropriate diagnostic indications for UGIE include: evaluation of an upper abdominal symptom that persists despite an appropriate trial of therapy, upper abdominal symptoms associated with alarm features that have been suggested as indicators of high risk for a serious disease [4, 5]. These features include recent onset of dyspepsia in an older patient, dysphagia, persistent vomiting, haematemesis/melena stools, anemia and/or weight loss. Other indications are diseases in which the presence of UGI pathology might modify the management (e.g., patients who have a history of ulcer or gastrointestinal bleeding who are scheduled for organ transplantation, long-term anticoagulant, or non-steroidal anti-inflammatory drug therapy for arthritis and those with cancer of the head and neck), familial adenomatous polyposis coli syndrome, suspected neoplastic lesions, peptic ulcer, upper gastrointestinal stricture or obstruction, gastrointestinal bleeding, caustic substance ingestion, and evaluation of chronic diarrhea among others [6, 7]. UGIE has been found to be both effective and a relatively safe procedure that can be performed at large medical centres, small rural hospitals, outpatient clinics or even private offices [8]. Establishing causes of UGI diseases leads to more efficient treatment and consequently decreases morbidity and mortality rates. In Ghana, UGIE service is offered in three teaching hospitals and a few other public or private centers, all in the cities. There are many reports in the literature on the indications and findings of UGIE mainly from the teaching hospitals and few private hospitals. However, scanty data are available from the districts hospitals on the profile of patients attending endoscopy unit for examination in this country. This study aims to document the demographic characteristics, indications and endoscopic findings of patients undergoing UGIE at a district hospital in Ghana.

## Methods

A formal approval of this study was obtained from the Ethical and Protocol Committee of the University of Ghana School of Medicine and Dentistry. This study was conducted in accordance with the Helsinki Declaration. The study used a cross-sectional design to consecutively recruit medical in-patients and clinic out-patients referred to the Endoscopy Unit of the St. Dominic Hospital (SDH) with UGI symptoms for endoscopy, from 14^th^ January, 2018 to 14^th^ December, 2018. SDH was founded in 1960 and has 339 beds and is the district hospital of Denkyembour district, Akwatia in the eastern region of Ghana and the main referral centre for other surrounding district hospitals. It offers a breadth of medical and surgical services including gastroenterology and endoscopy. Study participant recruitment and data collection was performed at the Endoscopy Unit, SDH, between January 2018 and December 2018. The Endoscopy Unit is manned by a medical gastroenterologist with the support of trained nurses and auxiliary staff. It uses Olympus and video endoscopy equipment for endoscopic procedures. It runs endoscopy sessions twice per week and offers both upper and lower Gastrointestinal (GI) endoscopy services. Each session performs approximately 5 upper endoscopies and 1 lower GI endoscopy. Procedures performed are both diagnostic and interventional. The latter include injection sclerotherapy and variceal band ligation.

Medical in-patients and clinic out-patients with gastrointestinal symptoms referred to the Endoscopy Unit, SDH were enrolled into the study. Study participants were consecutively recruited each week from endoscopy unit. All patients were given explanatory statements of the project and consented prior to endoscopy. Non-consenting patients were excluded from the analysis. Demographic data of the patients were taken including age, sex, occupation etc. Indications for the UGIE were recorded. UGIE was performed using the Olympus CV-160 videoscope. Study participants were given the option of sedation with (intravenous midazolam 2mg) or 10% lidocaine (xylocaine) throat spray. *H. pylori* infection was determined by the rapid-urease-campylobacter like- organism (CLO) test on gastric antral and body biopsies at UGIE (specificity 98%, sensitivity > 93%; Cambridge Life Sciences Ltd, Cambridge, UK). Endoscopic findings per each participant were recorded. Statistical analysis was performed using Stata 15^®^ statistical software package. Results were expressed as median and interquartile range for continuous variables and proportions for nominal variables. The proportion of the major endoscopic findings was presented on a 95% confidence interval.

## Results

**Figure 1:**
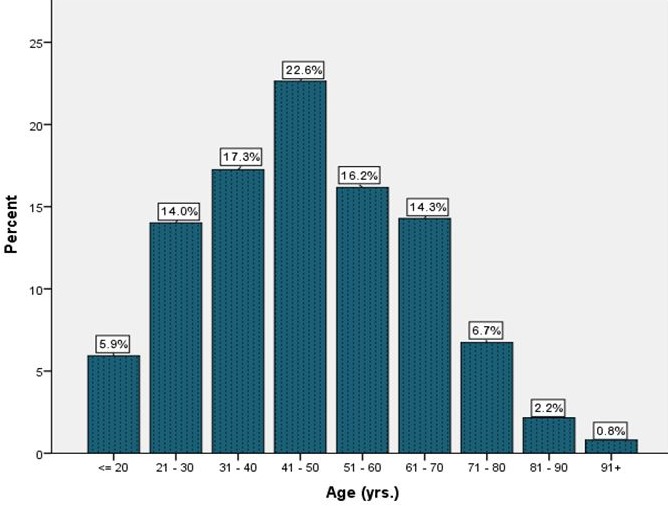
Age distribution of the study participants

There were 159(42.9%) males out of the 371 patients. Their ages ranged from 4 to 94 years with a median age of 46 years (Table 1). The 41-50 year age group had the highest frequency of 84(22.6%) patients, followed by the 31-40 year age group with 64(17.3%) patients. Other details of the age distribution are shown in Figure 1. Dyspepsia was the commonest indication occurring in 282(76.0%) patients followed by upper GI bleeding symptoms (hematemesis and melena stools) representing 70(18.9%) patients (Table 2). The major endoscopic diagnoses were gastritis which occurred in 261(70.4%) patients followed by duodenitis in 103(27.8%) patients, gastric ulcer (40/371, 10.8%), normal findings (30/371, 8.1%), duodenal ulcer (25/371, 6.7%) and oesophageal varices (15/371, 4.0%) (Table 3). The prevalence of *H. pylori* obtained by immediate CLO-testing of gastric antral and body biopsies for 356 patients out of 371 was 44.9% (Table 1). Amongst the 278 dyspeptic patients, gastritis was the commonest finding (212/278, 76.3%) followed by duodenitis (92/278, 33.2%) (Table 4).

**Table 1:** Demographic characteristics and Campylobacter-like organism (CLO test)

Age, Sex and CLO-Test	Frequency (%)
**Sex (n=371)**	
Male	159 (42.9)
Female	212 (57.1)
**Age (yrs.)**	
Overall	* 46 (33,60)
Males	*45 (34,58)
Females	*48 (31,61)
CLO TEST Positive (n=356)	160 (44.9)

* Median (interquartile range)

**Table 2:** Indications of endoscopy

Main indication for endoscopy	Frequency	Percent (Out of 371)
Dyspepsia	282	76.0
UGIB (melena/hematemesis)	70	18.9
Cirrhosis screen	10	2.7
Dysphagia	4	1.1
Vomiting	5	1.4
Portal hypertension	1	0.3
Epigastric mass	1	0.3
Odynophagia	1	0.3

*Multiple response analysis (total > 371)

**Table 3:** Primary endoscopy findings

Findings	Frequency (%)	95% CI
Gastritis	261 (70.4)	65.9 - 75.2
Duodenitis	103 (27.8)	23.5 - 32.7
Gastric ulcer	40 (10.8)	7.8 - 14.2
Normal	30 (8.1)	5.7 - 11.4
Duodenal ulcer	25 (6.7)	4.3 - 9.5
Esophageal varices	15 (4.0)	2.5 - 6.6
Esophagitis	9 (2.4)	1.2 - 4.6
Gastric cancer	6 (1.6)	0.7 - 3.5
Gastropathy	3 (0.8)	0.2 - 2.5
Gastric outlet obstruction	3 (0.8)	0.2 - 2.5
Esophageal cancer	2 (0.5)	0.1 - 2.2
Esophageal ulcer	2 (0.5)	0.1 - 2.2
Polyps	1 (0.3)	0.03 - 1.9
Esophageal stricture	1 (0.3)	0.03 - 1.9
Mallory-Weiss tear	1 (0.3)	0.03 - 1.9
Esophageal candidiasis	1 (0.3)	0.03 - 1.9

*Multiple response analysis (total > 371)

**Table 4:** Endoscopic findings of dyspeptic patients alone

Findings, dyspeptic patients (n=278)	Frequency (%)	95% CI
Gastritis	212 (76.3)	71.1 - 81.1
Duodenitis	92 (33.2)	27.8 - 39
Normal	24 (8.6)	5.9 - 12.6
Gastric ulcer	21 (7.5)	4.7 - 11
Duodenal ulcer	15 (5.4)	3.0 - 8.4
Esophagitis	6 (2.2)	0.9 - 4.7
Gastric cancer	2 (0.7)	0.1 - 2.8
Gastric outlet obstruction	1 (0.4)	0.05 - 2.5
Esophageal candidiasis	1 (0.4)	0.05 - 2.5

*Multiple response analysis (total >278)

## Discussion

This study aimed to document the demographic characteristics, indications and endoscopic findings of patients undergoing UGIE at the district hospital in Ghana. This study represents the first ever report on UGIE indications and findings from a district hospital in Ghana. Previously published work involving UGIE among Ghanaian patients have all come from the teaching hospitals and private hospitals in Accra, Kumasi and Tamale [9-13]. Dyspepsia was the commonest indication for upper GI endoscopy in the vast majority of our participants. This is similar to studies conducted in Ghana and other West African and East African countries [12, 14-17]. Other reasons for UGIE among our patients were symptoms of UGI bleeding, screening for oesophageal varices in cirrhotic patients and recurrent vomiting. Only 1.1% of our patients underwent upper GI endoscopy for dysphagia which is similar to 1.0% reported by study done in Kumasi, Ghana [12]. This differs from a study done in Malawi by Wolf *et al.* [18] which reported dysphagia as the most common indication for UGIE. Thirty seven percent (37%) of their patients had dysphagia as an indication for UGIE. The high prevalence of oesophageal cancer in Malawi may account for this difference [19]. Also endoscopy services are restricted in Malawi and as such only patients with alarm symptoms are referred for upper GI endoscopy [18].

Gastritis was the most frequent endoscopic finding in our patients, followed by duodenitis. This is comparable to previous Ghanaian studies [3, 10], which reported gastritis and duodenitis as common endoscopic findings among their patients. Gastric ulcer was diagnosed more frequently than duodenal ulcers among our patient population and this is similar to the study conducted by Gyedu *et al.* [12] in Kumasi. This is in contrast to the findings of one study from Accra that reported more duodenal ulcers than gastric ulcers [3]. This study was conducted mainly in a farming community and most of them may abuse non-steroidal anti-inflammatory drugs (NSAIDS) after farm work. Many of the patients in this study were in their middle age or older and probably on NSAIDS for degenerative joint and bone diseases which predispose more to the development of gastric ulcers. Gastric perforations were more common than duodenal perforations among the Kumasi population according to the study conducted by Ohene *et al.* [20]. They also noted that patients presenting with gastric perforations were more frequent users and abusers of NSAIDS and herbal medicines or concoctions [20]. The percentage of oesophageal varices detected in this study was more than previous studies published in this country [12]. This is because as part of the indications for endoscopy, patients with liver cirrhosis without bleeding were referred for endoscopy in this study. Normal findings in this study were far lower than earlier studies that have been published in this country [3, 12]. The difference may probably be due to improved endoscopic techniques in identifying UGI pathology or improve methods in clinical diagnosis over the decade or may be as a result of scarce availability of endoscopy services so people are referred appropriately for endoscopy. The use of Proton Pump Inhibitor (PPI) and NSAID could also modify the findings of endoscopy and information about this was not available in all the studies.

*H. pylori* colonization of the gut is one of the most common infections globally. Some researchers described it as the most common chronic human bacterial infection [21, 22]. It is the main cause of chronic gastritis and the principal etiological agent of gastric cancer and peptic ulcer disease. In many countries, the incidence of *H. pylori* has been decreasing in association with improved standard of living and improved portent antibiotics. The prevalence of *H. pylori* in this study was 44.9%. This is comparable to 45.2% reported by Darko *et al.* [13], but in contrast to 74.8% reported by Archampong *et al.* [23], in the country. Other previous studies in Ghana [24], Nigeria [25] and other developing countries [26] have also reported high prevalence of *H. pylori*. Possible reasons for this difference may be the increasing effective eradication therapy of the infection with antibiotic combination and proton pump inhibitors (PPI) and also the widespread and indiscriminate use of antibiotics and PPI. This study did not exclude patients who were already on antibiotics and PPI or have taken these drugs prior to the study. It may also be associated with improved sanitation among the inhabitants [27]. Despite the decrease in prevalence of *H. pylori* among patients in this study, the current prevalence of 45.2% is still high compared to rates in developed countries [28]. The prevalence of *H. Pylori* infection is associated with lower socioeconomic status, sanitation, basic hygiene, poor diet; overcrowding, ethnicity, gender and age, low levels of education and geographic location also play a major role in the distribution of the infection [29, 30]. This may explain the higher prevalence of *H. Pylori* in developing countries.

## Conclusion

The commonest indication for UGIE in the studied patients from a district hospital in Ghana was dyspepsia and most of these patients had gastritis on endoscopy. Only few patients had normal findings. Gastric ulcers were commoner than duodenal ulcers in this patient population. The prevalence of *H. pylori* in this population is low compared with most of the previous studies done in Ghana and other African countries. The outcomes of this study have implications for policy and planning. There is a need to identify the common causes of dyspepsia/gastritis in the community. This will help formulate and put in place community-based interventions including education to avoid these precipitating factors. There is also the need to establish more endoscopy centers in the district hospitals in this country and more health professionals trained to perform them.

### What is known about this topic

- In many developing countries, the *H. pylori* infection has a high prevalence rate of 80-95%;
- Patients with dyspepsia in the absence of any other alarm symptoms are more likely to have normal endoscopic findings.

### What this study adds

- The prevalence of *H. pylori* obtained in this district based study was 44.9%, far lower than prevalence of many studies conducted in Ghana and other Africa countries;
- Normal findings of patients with dyspepsia without any other symptoms in this study were far lower than earlier studies conducted on this subject.

## Competing interests

The authors declare no competing interests.
